# Functional Characterization of *PoEP1* in Regulating the Flowering Stage of Tree Peony

**DOI:** 10.3390/plants13121642

**Published:** 2024-06-14

**Authors:** Yang Lei, Jingshan Gao, Yuying Li, Chengwei Song, Qi Guo, Lili Guo, Xiaogai Hou

**Affiliations:** College of Agronomy/Tree Peony, Henan University of Science and Technology, Luoyang 471023, China; leiy_99@163.com (Y.L.); gjsssss2021@163.com (J.G.); lixueer593@126.com (Y.L.); songchengwei89@163.com (C.S.); guoqi0529@haust.edu.cn (Q.G.)

**Keywords:** tree peony, flowering regulation, *PoEP1* gene, functional analysis, expression analysis

## Abstract

The tree peony, a traditional flower in China, has a short and concentrated flowering period, restricting the development of the tree peony industry. To explore the molecular mechanism of tree peony flowering-stage regulation, *PoEP1*, which regulated the flowering period, was identified and cloned based on the transcriptome and degradome data of the early-flowering mutant *Paeonia ostii* ‘Fengdan’ (MU) and *Paeonia ostii* ‘Fengdan’ (FD). Through bioinformatics analysis, expression pattern analysis, and transgene function verification, the role of *PoEP1* in the regulation of tree peony flowering was explored. The open-reading frame of *PoEP1* is 1161 bp, encoding 386 amino acids, containing two conserved domains. *PoEP1* was homologous to the *EP1* of other species. Subcellular localization results showed that the protein was localized in the cell wall and that *PoEP1* expression was highest in the initial decay stage of the tree peony. The overexpression of *PoEP1* in transgenic plants advanced and shortened the flowering time, indicating that *PoEP1* overexpression promotes flowering and senescence and shorten the flowering time of plants. The results of this study provide a theoretical basis for exploring the role of *PoEP1* in the regulation of tree peony flowering.

## 1. Introduction

Tree peony (*Paeonia* section *Moutan* DC.), a precious traditional ornamental flower in China, has high ornamental, cultural, and economic value. The flowering time is one of the important indicators of the ornamental value of tree peony varieties; the tree peony typically flowers annually from mid-to-late April, spanning 50–60 days from bud to wilting. Each flower bloom lasts only 3–5 days, with group flowering lasting 10–15 days [1,2]. The short natural flowering period and excessive concentration of tree peony seriously affect the reputation and economic income of the tree peony production area [3]. An in-depth analysis of the molecular regulatory mechanisms related to tree peony flowering regulation, enabling it to complete the entire physiological process from flowering to senescence at an appropriate time. This will help to improve the growth development and reproductive capacity of the tree peony, meet the demand for the tree peony’s ornamental traits, greatly improve the ornamental value of tree peony, and bring economic benefits.

The transition from flowering to senescence is a crucial biological process in plant growth and development, playing a key role in reproduction and ecological adaptation. This process impacts various agronomic traits of crops, such as seed quantity and quality, fruit ripening, post-harvest ripening, and quality degradation, which determine their ornamental value and economic benefits [4]. Therefore, studying the molecular regulatory mechanisms during this transition in plants is essential for molecular genetic improvement breeding and post-harvest preservation. Regulating the transition from flowering to senescence is part of flower-stage regulation. Controlling the plant’s flowering period helps maintain high quality, avoid unfavorable environments, increase yield, and maximize economic benefits. The genetic mechanism of *Arabidopsis thaliana* flowering reveals that higher plants regulate flowering through six pathways: photoperiodism, gibberellin signaling pathway (GA), vernalization response pathway (VRN), autonomous pathway (AUT), age-related pathway (AGE), and temperature-dependent pathway (TEMP) [5,6,7]. These pathways are interdependent yet interconnected with each other to form a complex network regulatory system [8].

In recent years, researchers have made significant progress in unraveling the genetic mechanisms of flowering and senescence regulation in *Arabidopsis thaliana*, with the identification of numerous genes that regulate the flowering stage through both forward and reverse genetics approaches. *CONSTANS* (*CO*), a crucial floral promoter downstream of the photoperiod pathway, functions as a key inducer of flowering. The expression of *CO* messenger ribonucleic acid (mRNA) induces flowering when it aligns with plant exposure to light [9]. *FLOWERING LOCUS C* (*FLC*) serves as a convergence point in the autonomous and vernalization pathways, exerting regulatory roles in multiple flowering control pathways. Overexpression of *FLC* can inhibit flowering through autonomous and vernalization pathways [10,11]. Integrator factors of the flowering pathway, located downstream of *CO* and *FLC*, activate or inhibit plant flowering signals, precisely regulating the expression of floral meristem genes and controlling the flowering and senescence process [12]. Studies have also shown that microRNAs establish an intrinsic regulatory network that drives plant flowering through the modulation of target genes. The miR156/SPL pathway has emerged as a novel age-dependent flowering regulatory pathway. miR156 negatively regulates *SQUAMOSA-PROMOTER BINDING PROTEIN-LIKE* (*SPL*) genes to delay flowering in plants [13]. *SPL* defines a separate endogenous flowering pathway, with high levels of miR156 preventing early flowering in plants [14,15]. In addition, studies have demonstrated that the miR156-*SPL*-miR172 signaling cascade in *Arabidopsis thaliana* is a crucial determinant that controls the transition from the juvenile phase to the adult phase, as well as the reproductive growth period in plants. Furthermore, the expression of miR172 is regulated by environmental temperature and the photoperiod, with different miR172 variants exhibiting distinct response patterns to control the expression of the corresponding target genes and initiate plant flowering [16,17]. *VERNALIZATION INSENSITIVE 3* (*VIN3*) is a key gene in the vernalization pathway that is capable of sensing low temperatures over time. *PoVIN3* recognizes the duration of low-temperature treatment during vernalization and works in conjunction with *VRN1*, *VRN2*, and *VRN5* at low temperatures, accelerating flowering by inhibiting the *FLC* gene. This complex interplay of genes and environmental cues ultimately influences the timing of flowering to senescence in plants [18]. Furthermore, the *SHORT VEGETATIVE PHASE* (*SVP*) gene of *Paeonia lactiflora* negatively regulates the flowering stage of plants; it is suggested that it exerts its negative regulatory function by inhibiting the expression of *FLOWERING LOCUS T* (*FT*), *SUPPRESSOR* OF *OVEREXPRESSION* OF *CO1* (*SOC1*), *LEAFY* (*LFY*), *APETALA1* (*AP1*), and other genes [19]. FLOWERING LOCUS M (FLM) is an important flowering inhibitor; it mainly inhibits flowering under low temperature conditions but has no inhibitory effect or a weak inhibitory effect under high temperature conditions [20,21,22,23]. *Senescence-Related Gene* (*SRG1*) may promote the synthesis of assimilates in plants through the GA pathway, thereby improving the growth and flowering of transgenic *Arabidopsis thaliana* and delaying senescence [24].

The *EP1* gene (*epithelial-specific secreted glycoprotein 1*) encodes a glycoprotein that is specifically secreted by epithelial cells and plays an important role in maintaining the structure and function of epithelial cells. At present, there are few reports in the literature on the *EP1* gene. *EP1* may regulate plant growth, development processes, and responses to stress. Such previous reports indicate that *EP1* plays a certain role in the resistance of cotton to drought stress. Up-regulation of *EP1* leads to an increase in MG synthesis, activating the glyoxase and peroxidase systems to enhance plant drought resistance [25]. However, there is nothing in the literature on the function and role of *EP1* in regulating plants’ flowering stage. More research is needed to confirm its exact role. In this study, we identified *PoEP1*, a gene potentially associated with regulating the flowering stage of the tree peony, through an analysis of transcriptome and degradome data conducted in the laboratory. The cloning of *PoEP1* in the tree peony using molecular biology techniques marks the first instance of such an achievement. Subsequently, the gene’s structure underwent a preliminary bioinformatics analysis, which was followed by a detailed examination of its expression pattern and function to explore the characteristics of *PoEP1* and its role in regulating tree peony flowering. The comprehensive investigation aimed to establish a theoretical and empirical foundation for understanding the mechanism underlying tree peony flowering regulation.

## 2. Results

### 2.1. Expression Analysis of PoEP1 in the Petals of MU and FD at Different Flowering Stages

**Figure 1:**
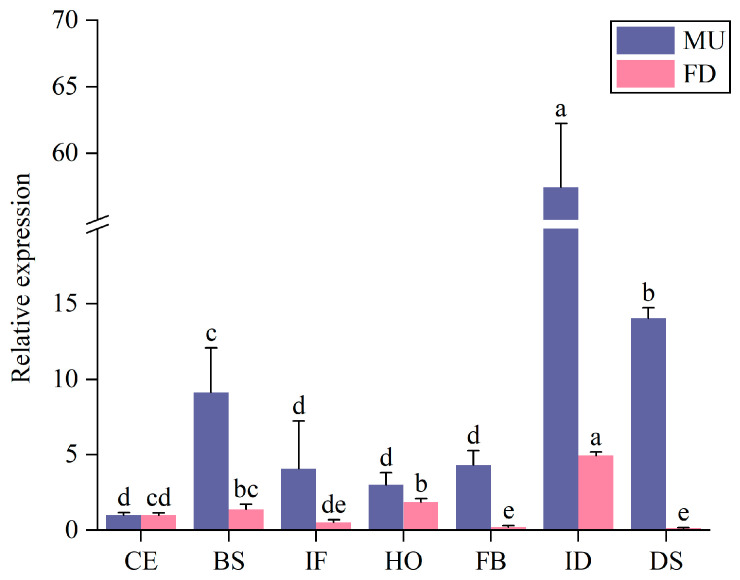
Relative expression levels of *PoEP1* in different flowering stages of MU and FD. CE: color-exposure stage, BS: blooming stage, IF: initial flowering stage, HO: half opening stage, FB: full blooming stage, ID: initial decay stage, DS: decay stage. MU: mutant *Paeonia ostii* ‘Fengdan’. FD: *Paeonia ostii* ‘Fengdan’. Different lowercase letters (a–e) indicate significant differences (*p* < 0.05).

To investigate the expression patterns of *PoEP1* in MU and FD across different flowering stages, we utilized real-time fluorescence quantitative technology. The analysis focused on examining the expression levels of *PoEP1* in the petals at seven distinct flower developmental stages. These stages encompassed the color-exposure stage (CE), blooming stage (BS), initial flowering stage (IF), half-opening stage (HO), full-blooming stage (FB), initial decay stage (ID), and decay stage (DS). The study samples consisted of petals from the early-flowering mutant *Paeonia ostii* ‘Fengdan’ (MU), and *Paeonia ostii* ‘Fengdan’ (FD). The results showed that *PoEP1* was expressed in MU and FD. The expression levels of *PoEP1* in the flowering stages of the MU and FD reached a higher level in the ID stage, indicating that *PoEP1* mainly plays a role in the ID stage of the tree peony (Figure 1).

### 2.2. Construction of Overexpression Vector for PoEP1

**Figure 2:**
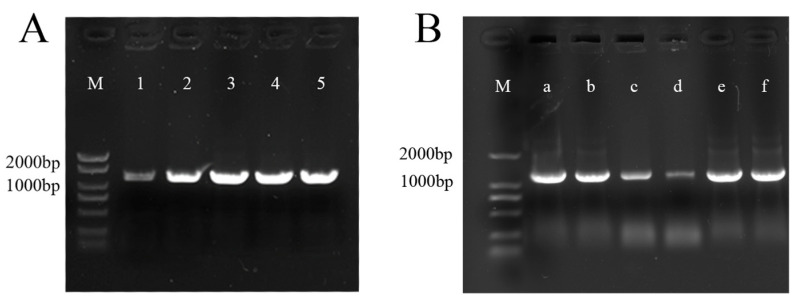
PCR amplification of *PoEP1*. (**A**) Electrophoretic map of cloned *PoEP1*. M is marker DL2000+, and 1–5 are amplification products of *PoEP1.* (**B**) PCR detection of *Agrobacterium tumefaciens* pCAMBIA2300-*PoEP1*. M is marker DL2000, and a–f are amplification products of pCAMBIA2300-*PoEP1*.

The cDNA of FD was used as a template for PCR amplification, and the 1161 bp specific band obtained after gel electrophoresis was named *POEP1* (Figure 2A). The gene sequence was submitted to GenBank (PP496090) in NCBI. The fragments were recovered, linked to the cloning vector pTOPO-AT, transformed into competent *E. coli* DH5α, and sequenced. The sequencing results were analyzed by DNAMAN alignment, and it was found that the obtained fragments were consistent with the original sequences, indicating that the *PoEP1* sequence had been successfully cloned. The overexpression vector pCAMBIA2300 was subjected to single digestion with *Sal* I, and the linearized pCAMBIA2300 was recovered by cutting the gel. The above-cloned correct pTOPO-*PoEP1* recombinant plasmid was amplified with specific primers to *Sal* I. The single-enzyme cleavage site was cut to recover the *PoEP1* gene fragment, which was inserted into the expression vector pCAMBIA2300 and transformed with *E. coli* DH5α. Positive transformants were preliminarily identified by colony PCR. The 1161 bp *PoEP1* fragment was sent for sequencing. The sequencing results were consistent with the original sequence, indicating that the *PoEP1* gene overexpression vector was successfully constructed, which was named pCAMBIA2300-*PoEP1*. pCAMBIA2300-*PoEP1* was transformed into the *Agrobacterium tumefaciens* strain GV3101. Monoclonal colonies were screened and verified by PCR with specific primers (Figure 2B), and a band of 1161 bp was obtained, indicating that the *PoEP1* gene had been successfully introduced into *Agrobacterium tumefaciens* and could be used for transformation.

### 2.3. Bioinformatics Analysis of PoEP1

**Figure 3:**
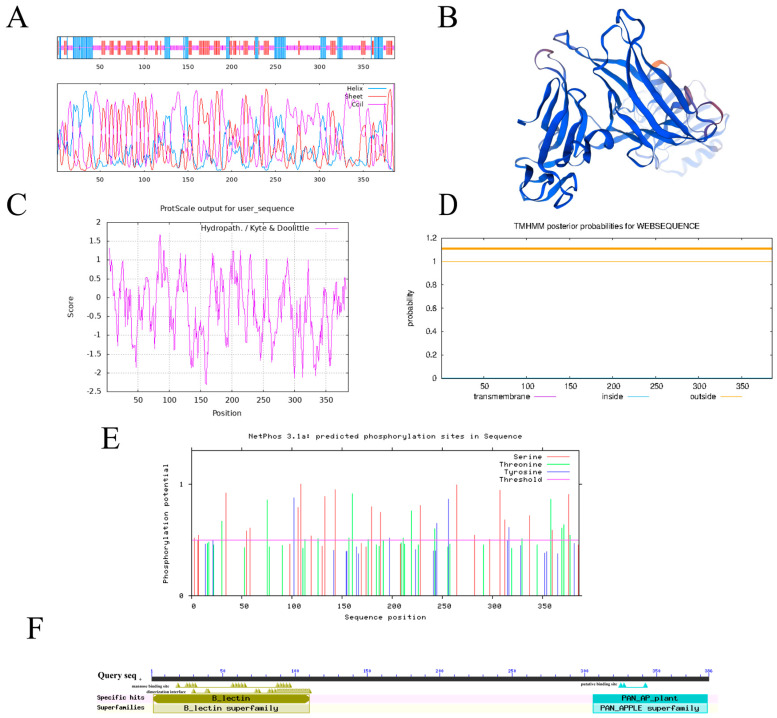
Bioinformatics analysis of *PoEP1*. (**A**) Secondary structure prediction of the *PoEP1*-encoded protein. (**B**) Tertiary structure prediction of the *PoEP1*-encoded protein. (**C**) Hydrophilic analysis of the *PoEP1*-encoded protein. (**D**) Analysis of the *PoEP1* transmembrane domain. (**E**) Analysis of phosphorylation sites of the *PoEP1*-encoded protein. (**F**) Conservative domain prediction.

Bioinformatics analysis of the *PoEP1* gene sequence showed that the open reading frame (ORF) length of the *PoEP1* gene was 1161 bp, encoding 386 amino acids (Figure S1). The molecular weight of the protein was 43.2 kDa. The theoretical isoelectric point pI was 7.45, and the instability index was 34.50, indicating that the protein encoded by *PoEP1* was a stable protein. The results of secondary structure prediction showed that the protein encoded by *PoEP1* accounted for 20.98% α helix, 26.68% extended chain, and 52.33% random coil (Figure 3A). The three-dimensional structure suggests that the protein is involved in the regulation of cellular action in the form of monomers (Figure 3B). The prediction results of hydrophilicity and hydrophobicity showed that the protein encoded by *PoEP1* was a hydrophilic protein (Figure 3C). TMHMM analysis showed the absence of a transmembrane structure in this protein (Figure 3D). Phosphorylation site prediction showed that the protein had 21 serine, 15 threonine, and 4 tyrosine residues (Figure 3E). The analysis of conserved domains showed that the protein contained two conserved domains: B-lectin (bulb-type mannose-specific lectin), which contains a three-fold internal repeat, and PAN-AP-plant (plant PAN/APPLE-like domain), which is present in plant S-receptor protein kinases and secreted glycoproteins (Figure 3F).

**Figure 4:**
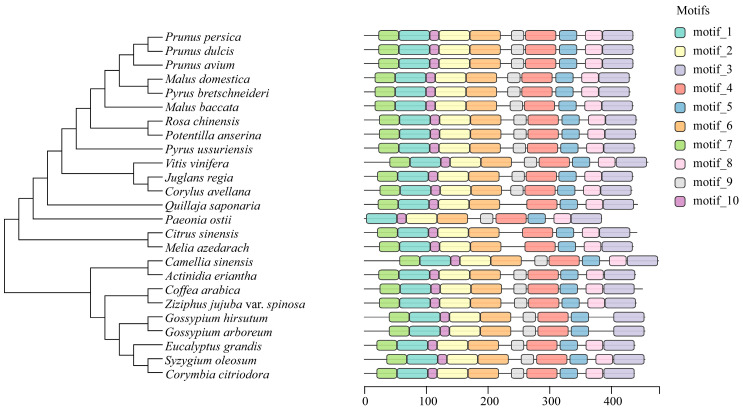
Phylogenetic tree and motif analysis of the PoEP1. The left side shows the phylogenetic tree, and the right side displays the analysis of conserved protein regions with different colors representing various motif regions.

**Figure 5:**
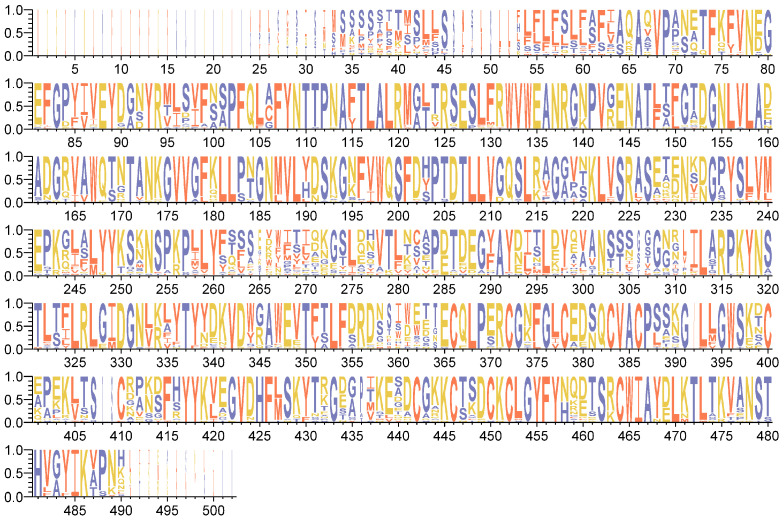
Multiple sequence comparison using WebLogo. The height of each letter in the figure corresponds to the frequency of the amino acid at that location; the letters at each position are arranged from most conservative to least conservative.

To explore the evolutionary relationship between *PoEP1* in the tree peony and *EP1* in other plants, the amino acid sequences encoded by *PoEP1* obtained by cloning were searched for in the NCBI database, and a total of 24 species of *EP1* gene-encoded amino acid sequences were obtained for phylogenetic tree analysis (Figure 4). The results showed that the protein encoded by *PoEP1* has a closer genetic relationship with *Citrus sinensis* and *Melia azedarach*, and has a farther genetic relationship from *Corymbia citriodora*, *Syzygium oleosum*, and *Eucalyptus grandis*. The results of the protein-conserved region analysis showed that the conserved region of *PoEP1* in tree peony was the same as that of other species, and the protein encoded by *PoEP1* in the tree peony had one less region than that of other species (Figure 4). Multi-sequence alignment analysis of the protein encoded by *EP1* and the protein encoded by *PoEP1* in these 24 species showed that most of the bases were highly conserved, with some sites even identical and only a few sites with large base changes (Figure 5).

### 2.4. Subcellular Localization of PoEP1

**Figure 6:**
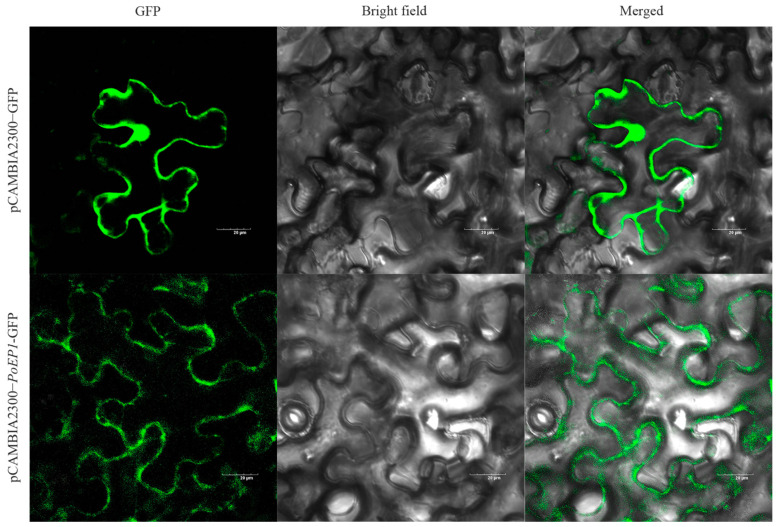
Subcellular localization of PoEP1. pCAMBIA2300-GFP is the control group; pCAMBIA2300-*PoEP1*-GFP is the experimental group.

The results of the subcellular localization prediction showed that the protein encoded by *PoEP1* was located in the cell wall. The fusion expression vector pCAMBIA2300-*PoEP1*-GFP was transiently transformed in tobacco to determine the subcellular localization of the protein encoded by *PoEP1*, and the pCAMBIA2300-GFP empty vector was used as a control. Subsequently, the infected tobacco leaves were observed by laser confocal scanning microscopy. The transient transformation system localization results (Figure 6) showed that the pCAMBIA2300-GFP empty vector distributed green fluorescence in all parts of the cell under the excitation at 488 nm. pCAMBIA2300-*PoEP1*-GFP also showed green fluorescence in the cell wall, which was consistent with the predicted results, indicating that the protein encoded by the *PoEP1* gene was localized to the cell wall.

### 2.5. Identification and Phenotypic Observation of Transgenic Arabidopsis thaliana

**Figure 7:**
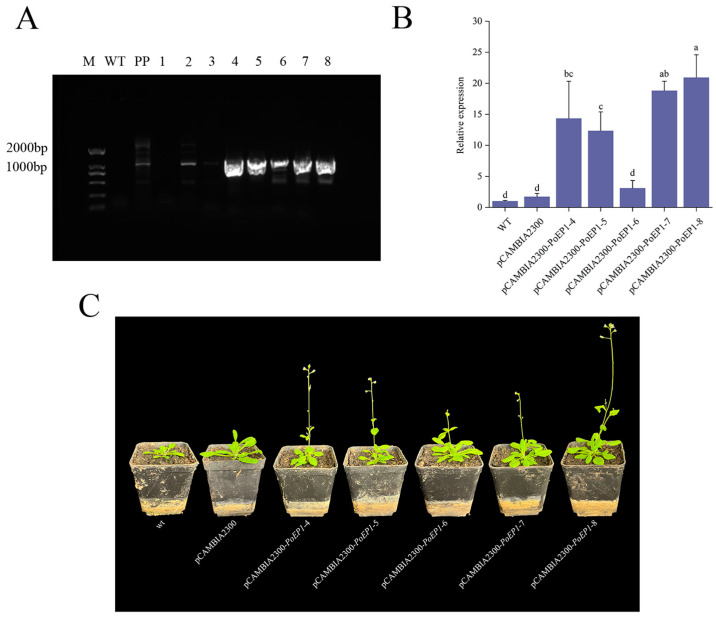
The phenotype and PCR amplification of *Arabidopsis thaliana* transformed with *PoEP1*. (**A**) PCR identification of transgenic *Arabidopsis*. M: marker DL2000, WT: wild-type *Arabidopsis*, PP: positive plasmid, 1–8: transgenic *Arabidopsis*. (**B**) Relative expression of *PoEP1* in transgenic lines and wild *Arabidopsis*. Different lowercase letters (a–d) indicate significant differences at *p* < 0.05. (**C**) Phenotypic observation of transgenic *Arabidopsis*.

The transgenic *Arabidopsis thaliana* plants were cultured to the T3 generation. Subsequently, eight transgenic *Arabidopsis thaliana* plants were selected for observing the flowering stage and collecting samples. The transgenic *Arabidopsis thaliana* plants were confirmed via genomic DNA PCR, with wild-type *Arabidopsis thaliana* and the pCAMBIA2300-*PoEP1* plasmid serving as negative control and positive control, respectively. Notably, wild-type *Arabidopsis* did not exhibit the target bands, while the positive plasmid and the transgenic lines 2 and 4–8 displayed correct bands, signifying that *PoEP1* was successfully integrated into *Arabidopsis* (Figure 7A). The fourth, fifth, sixth, seventh, and eighth transgenic positive *Arabidopsis* plants were selected for real-time fluorescence quantitative analysis, and flowering-related phenotypes were observed. The qRT-PCR results showed the expression of *PoEP1* was significantly upregulated in transgenic lines compared with WT and pCAMBIA2300 (Figure 7B). The transgenic plants flowered approximately 5–7 days earlier than both the WT control group and the pCAMBIA2300 control group (Figure 7C). Additionally, the flowering duration was significantly reduced, and the plants entered the decay phase earlier. It is inferred that *PoEP1* has the capability to promote flowering and abbreviate the flowering period in plants.

### 2.6. Analysis of the Expression Pattern of PoEP1 Overexpression on Tree Peony

**Figure 8:**
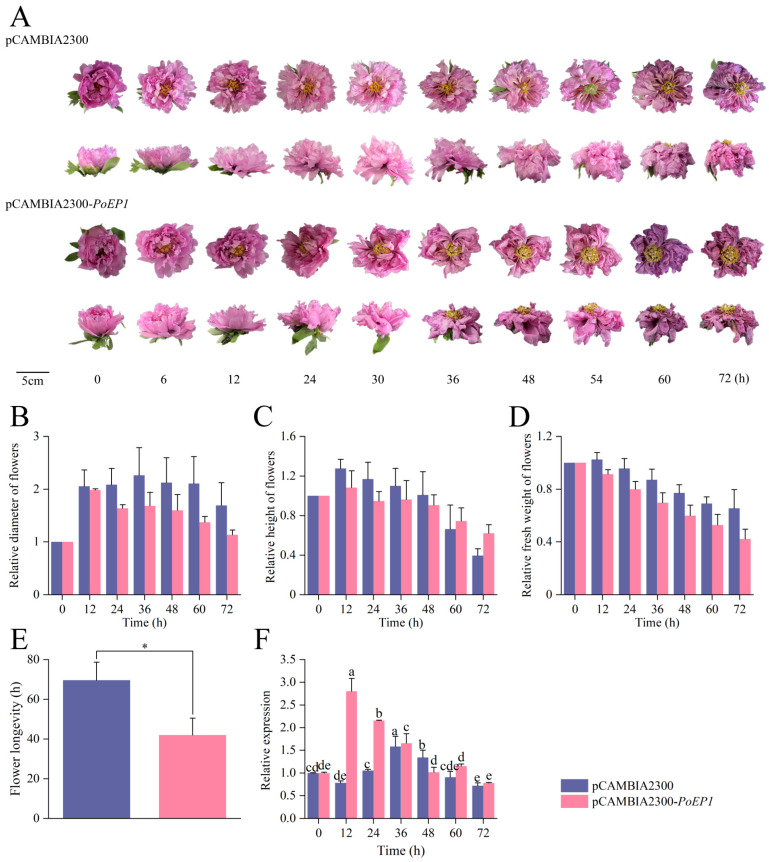
Transient overexpression of *PoEP1* in cut tree peony flowers. Tree peony fresh-cut flowers were infiltrated with *Agrobacterium tumefaciens* containing pCAMBIA2300 control and pCAMBIA2300-*PoEP1*. (**A**) Phenotype. (**B**) Relative diameter. (**C**) Relative height. (**D**) Relative fresh weight. (**E**) Flower longevity. (**F**) Relative expression of the empty vector pCAMBIA2300 and relative expression of the pCAMBIA2300-*PoEP1* in fresh-cut flowers. Error bars indicate standard error (SE). Different lowercase letters (a–e) and * indicate significant differences at *p* < 0.05.

To explore the possible expression and role of *PoEP1* in the tree peony, we used a transient overexpression method to overexpress *PoEP1* in fresh-cut flowers of the tree peony. *Paeonia ostii* ‘Fengdan’ is a single-petal variety, with fewer petals and a short flowering period, making it unsuitable for infection and observation as a fresh-cut tree peony flower. On the other hand, *Paeonia suffruticosa* ‘Luoyanghong’, which is a typical fresh-cut tree peony variety, was commonly used for studying various cut flowers of the tree peony. Therefore, ‘Luoyanghong’ was chosen as the subject for homologous infection in this study. Fresh-cut flowers of the CE stage in ‘Luoyanghong’ were used as the infection objects, with observation and recording after cultivation under appropriate conditions. The results indicate that pCAMBIA2300 fresh-cut flower plants reached IF at 0 h, HO at 6 h, FB at 12 h, ID at 54 h, and DS at 72 h. In contrast, pCAMBIA2300-*PoEP1* fresh-cut flower plants reached IF at 0 h, FB at 6 h, ID at 36 h, and DS at 60 h. The fresh-cut flowers infected with pCAMBIA2300-*PoEP1* reached FB 6 h earlier and DS 12 h earlier than the fresh-cut flowers infected with the empty vector pCAMBIA2300 (Figure 8A). The results suggest that *PoEP1* may have advanced and shortened the flowering time of the tree peony. There was no significant difference in the relative flower diameter (Figure 8B), relative flower height (Figure 8C), and relative fresh weight (Figure 8D) between the two, and the relative fresh weight showed a trend of first increasing and then decreasing. In addition, the average flowering duration for pCAMBIA2300 is about 69 h; pCAMBIA2300-*PoEP1* averages 42 h. The flowering time was significantly shortened by approximately 27 h compared with the empty vector (Figure 8E) (*p* < 0.05). The expression level of *PoEP1* in fresh-cut flowers infected with empty vectors reached the highest level at 36 h and, then, continued to decrease (Figure 8F). However, the relative expression of *PoEP1* in infected fresh-cut flowers reached the highest level 12 h after overexpression and, then, continued to decrease; the *PoEP1* was still expressed at 72 h (Figure 8F). The results showed that the *PoEP1* gene was overexpressed earlier in the fresh-cut flowers infected with pCAMBIA2300-*PoEP1* than with the empty vector pCAMBIA2300, and the expression level was higher than that of the empty vector.

## 3. Discussion

The transition time of vegetative growth to reproductive growth and the time of senescence and nutrient transport played important roles in plant yield and quality. The transition of plants from the vegetative stage to the reproductive stage is crucially marked by flowering, a quantitative trait regulated by multiple genes. Flowering to senescence is determined by a combination of genetic factors and environmental conditions, shaping a vital role in the reproduction and ecological adaptation of offspring [26]. In this study, the regulatory mechanisms influencing the flowering period of the tree peony were investigated using MU and FD as experimental materials. Flowering time is a crucial agronomic trait impacting crop yield, quality, and the ornamental and economic value of flowers. To identify genes crucial for tree peony flowering regulation, we leveraged our full-length transcriptome and degradome database established in our laboratory. Through molecular biology techniques, we successfully isolated and characterized *PoEP1* from the tree peony, providing novel insights into its expression patterns and functions.

Sequence analysis revealed that the *PoEP1* gene had a closer genetic relationship with *Citrus sinensis* and *Melia azedarach* and has a farther genetic relationship with *Corymbia citriodora*, *Syzygium oleosum*, and *Eucalyptus grandis*. The *PoEP1* gene contains two domains, namely the B-lectin and the PAN-AP-plant superfamily. B-lectin is a globular mannose-specific lectin that is widely distributed in higher plants and is thought to play a role in identifying high-mannose-based glycans from exotic microorganisms or plant predators. Mannose-specific lectin structural studies have shown that this domain contains a triple internal repeat (β-prism architecture), and binding specificity is mediated by different structural scaffolds [27,28]. The consensus sequence motif QXDXNXVXY is involved in α-D-mannose recognition, and lectins are carbohydrate-binding proteins that can specifically recognize a variety of carbohydrates and mediate a variety of biological processes [29]. It has been proven that mannose-binding proteins (MBPs) are a class of plant lectins containing the B-lectin domain that participate in a variety of physiological processes through binding to sugar, including cell-to-cell interactions and interactions between hosts and pathogens, etc. Proteins containing B-lectin domains can be divided into two groups. MdMBP2 belongs to class I, which, compared with class II proteins, lack the SLP domain and protein kinase domain that are involved in the apple’s defense response to the ringed disease pathogen and may play a role in host recognition of the pathogen [30]. The plant PAN-AP-plant domain is present in plant S-receptor protein kinases and secreted glycoproteins. This domain achieves a variety of biological functions by mediating protein–protein or protein–carbohydrate interactions. Furthermore, S-receptor protein kinases and S-site glycoproteins are involved in sporophyte self-affinity responses in *Brassica*, which may be one of the many molecular mechanisms by which hermaphroditic flowering plants avoid auto-fertilization [31].

According to the transcriptome and degradome data of tree peony in the early stage of flowering, the Kyoto Encyclopedia of Genes and Genomes (KEGG) enrichment analysis showed that the PoEP1 protein, with lectin-receptor-like kinases (LecRLKs), participates in the regulation of plant growth and development. LecRLKs are a subfamily of receptor-like kinases (RLKs). According to the different domains, lectin-receptor kinases can be divided into three types: L, G, and C [32,33]. Lectin proteins play a very important role in the whole life course of plants, including cell-to-cell communication, plant development, and defense responses. In recent years, an increasing number of studies have found that lectin-receptor kinases are involved in biotic/abiotic stress responses, plant disease-resistance responses caused by bacteria, fungi, herbivorous insects, etc., and in the regulation of plant growth and development. In rice, the lectin-receptor kinase OsLecRK is involved in the regulation of seed germination and disease resistance. Knockout of *OsLecRK* inhibits the expression of disease-related genes, thereby impacting plant resistance to bacteria, fungi, and herbivores [34]. *PWL1* encodes a G-type LecRLK with active kinase and autophosphorylation activity, which plays multiple roles in the regulation of plant growth and development, heat tolerance, and resistance to bacterial pathogens, and is also involved in the regulation of multiple biological processes, such as carbon metabolism, the ribosome, and peroxisome pathways. The expression of *PWL1* in rice was mainly observed in tillering and mature leaves, and a *PWL1* mutant enhanced the heat sensitivity and resistance of rice to white leaf blight and bacterial leaf spot [35]. *LecRK-VIII.2* plays a crucial role in *Arabidopsis thaliana* by regulating the growth of rosette leaves, roots, and stems through the coordination of the source–sink relationship. Furthermore, it demonstrates the ability to control the expansion and proliferation of seed-coat cells, thereby influencing seed size and yield. Research findings show that seeds that overexpress *LecRK-VIII.2* exhibit larger sizes but lower amounts of stakes and seeds, ultimately leading to similar yields as wild-type plants. On the contrary, mutants lacking *LecRK-VIII.2* display increased horn and seed production, resulting in higher seed yields. These results hold significant implications for crop genetics and breeding, as they shed light on the regulatory mechanisms impacting seed development and yield [36].

The results of the subcellular localization indicate that the protein encoded by the *PoEP1* gene was localized to the cell wall. The expression analysis of *PoEP1* showed that the expression trend varied among the MU and FD. Overall, expression was higher in MU than in FD in the flowering stage, and the expression level reached the highest in the initial decay stage. These results indicate that the gene has a certain variety specificity and is mainly expressed in large quantities when the flowers begin to decay, and these results provided ideas for the subsequent study of this gene.

The study of gene function in the tree peony is constrained by the low efficiency of genetic transformation, and the identification of functional genes in the tree peony depends on model plants such as *Arabidopsis thaliana*. The transient expression system is an effective means to quickly analyze gene function, and compared with the previous heterologous stable transformation, the homologous transient expression system established by the *Agrobacterium tumefaciens*-mediated method in plants can overexpress or silence the expression of the target gene in plant cells in a short time. This method has the advantages of simplicity, rapidity, efficiency, and accuracy, which can be used to evaluate the function of the target gene and the expected phenotype of transgenic plants in an early manner. Furthermore, the transient transformation efficiency can sometimes reach 1000 times that of stable transformants [37]. In recent years, many plant leaves, fruits, petals, roots, suspension cells, cell embryos, and calli have also been gradually used in the study of transient expression systems, which have been successfully used in phalaenopsis [38], roses [39,40], grapes [41], strawberries [42], carnations [43], and cassava [44]. These systems provide technical support for gene-function research. In this study, *Agrobacterium tumefaciens* was used to establish a transient transformation system of tree peony overexpression. We constructed the overexpression vector pCAMBIA2300-*PoEP1* and transiently transformed it into fresh-cut flowers of the tree peony. The results showed that, compared with the fresh-cut flowers infected with empty vectors, the fresh-cut flowers infected with pCAMBIA2300-*PoEP1* were overexpressed in advance. The flowering time was 6 h earlier. The decay time was 12 h earlier, and the flowering time was significantly shortened by 20 h (*p* < 0.05). The results of this study indicate that *PoEP1* can regulate the tree peony, resulting in it blooming earlier and shortening the flowering time.

In conclusion, the result indicated that *PoEP1* could advance plants’ flowering and senescence and shorten the flowering time of plants, providing a basis for verifying the function of *PoEP1* in the tree peony and providing research ideas and a technical basis for verifying other important functional genes in the tree peony.

## 4. Materials and Methods

### 4.1. Plant Materials

**Figure 9:**
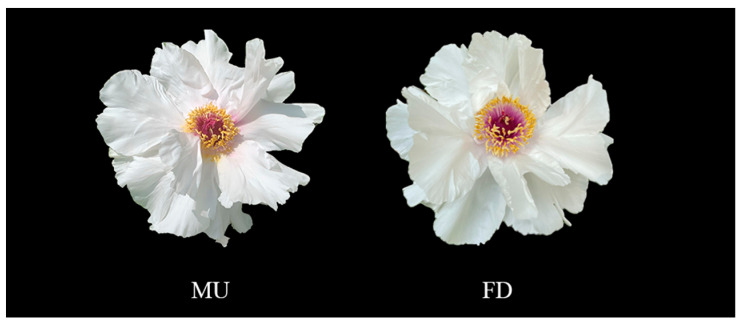
Samples of flowers of the tree peony MU and FD. MU: mutant of *Paeonia ostii* ‘Fengdan’. FD: *Paeonia ostii* ‘Fengdan’.

In this experiment, the mutant *Paeonia ostii* ‘Fengdan’ (MU) and *Paeonia ostii* ‘Fengdan’ (FD) were used as materials and planted in the germplasm nursery at Henan University of Science and Technology (112°24′52.05″ E, 34°35′45.91″ N). FD is an early-flowering variety, The flower is a single-petal type, totaling 10–15 pieces. The petals are broad and flat, mostly jade white but sometimes pink (Figure 9). It blooms in April and has a short flowering period. While MU is an early-flowering mutant line of FD obtained by selection from FD plants of natural mutation, there is essentially no difference in morphological characteristics between it and FD, but MU exhibited an earlier flowering phenotype compared to FD.

Samples were collected from different flower developmental stages of tree peony, frozen in liquid nitrogen, and stored at −80 °C for future use. These different flower developmental stages included the color-exposure stage (CE), blooming stage (BS), initial flowering stage (IF), half-opening stage (HO), full-blooming stage (FB), initial decay stage (ID), decay stage (DE).

### 4.2. RNA Extraction and Expression Analysis

An RNAprep Pure Plant Plus Kit (TianGen Biotech, Beijing, China) was used to extract total RNA at seven different flower developmental stages in MU, FD. The total RNA was reverse transcribed into cDNA using a two-step method. Specific quantitative primers were designed based on *PoEP1* gene sequences (Table 1), which were synthesized by Sangon (Sangon biotech, Shanghai, China). *EF1α* was used as the internal reference gene for quantitative analysis. According to the SYBR kit (Accurate Biology, Changsha, China), real-time quantitative PCR (qRT-PCR) was used to detect the relative mRNA expression. The quantitative system was as follows: 2 μL of cDNA template, upstream and downstream primers (10 μmol·L^−1^; 0.4 μL each), 2× SYBR Green Pro Taq HS Premix (10 μL), and RNase-free water (7.2 μL). The PCR amplification procedure was as follows: denaturation at 95 °C for 30 s, followed by 95 °C for 5 s and 60 °C for 30 s for 40 cycles. The relative mRNA expression level of *PoEP1* was calculated using the 2^−ΔΔCt^ method [1].

### 4.3. Cloning and Identification of the PoEP1 Gene Sequence in Tree Peony RNA

The primer for *PoEP1* gene cloning (Table 1) was designed using the software Primer 5.0, and the *PoEP1* gene was cloned using FD cDNA as the template. *PoEP1* was amplified by PCR with *TransStart^®^FastPfu* DNA Polymerase (TransGen Biotech, Beijing, China) (pre-denaturation at 95 °C for 2min; denaturation at 95 °C for 20s, annealing at 50 °C for 20 s, and extension at 72 °C for 30 s for 35 cycles; extension at 72 °C for 5 min), and the product was recovered using a gel-cutting and purification kit (TianGen Biotech, Beijing, China). After ligation with the pTOPO-TA vector (Mei5bio, Beijing, China), the recombinant plasmid was transferred into competent *E. coli* DH5α (Accurate Biology, Changsha, China). Monoclonal colonies were selected, and positive clones were identified by PCR, and sent to Sangon for sequencing verification.

### 4.4. Bioinformatics Analysis of PoEP1 in Tree Peony

The amino acid composition of *PoEP1*-encoded protein was analyzed by the online software Translate (http://web.expasy.org/translate/, accessed on 6 November 2023). The physicochemical properties of the protein encoded by *PoEP1* were analyzed by the online software ExPASy 3.0 (https://web.expasy.org/protparam/, accessed on 6 November 2023). The protein secondary structure prediction was assessed with SPOMA (https://npsa-prabi.ibcp.f/cgi-bin/secpredsopma.pl, accessed on 6 November 2023). The prediction of the protein’s 3D model was by SWISS-MODE (https://www.swissmodel.expasy.org/interactive, accessed on 6 November 2023), and the prediction of protein hydrophilicity and hydrophobicity was performed by Protscale (https://web.expasy.org/protscale/, accessed on 6 November 2023). The prediction of the transmembrane structure was performed by TMHMM 2.0 (http://www.cbs.dtu.dk/services/TMHMM, accessed on 6 November 2023). Phosphorylation site analysis was also performed by NetPhos 3.1 (http://www.cbs.dtu.dk/services/NetPhos/, accessed on 6 November 2023). The conserved domain was analyzed using CDD in the NCBI database (http://www.ncbi.nlm.nih.gov/Structure/cdd/wrpsb.cgi, accessed on 6 November 2023). The amino acid sequences encoded by *PoEP1* were blast-aligned in the NCBI database (http://blast.ncbi.nlm.nih.gov/, accessed on 6 November 2023). The amino acid sequences of different species were downloaded, and the phylogenetic tree was constructed using MEGA 6.0 software. Protein-sequence conserved-site analysis was performed using the online software MEME 5.5.5 (https://meme-suite.org/meme/tools/meme, accessed on 6 November 2023). The ClustalX 2.1 software was used to perform multi-sequence alignment of *PoEP1* sequences, and the alignment results were input into the online software WebLogo 3.7.12 (http://www.weblogo.berkeley.edu/logo.cgi, accessed on 6 November 2023) to analyze the conserved bases at each position. The results were plotted as Logo diagrams.

### 4.5. Construction of the PoEP1 Gene Overexpression Vector

The vector pCAMBIA2300 was subjected to single-enzyme digestion with the restriction enzyme *Sal* I (TaKaRa, Dalian, China), and the enzyme digest product was purified and recovered after electrophoresis. Gene-specific primers with *Sal* I digestion sites were designed, and the plasmids with the correct clone and sequencing were amplified by PCR to amplify the *PoEP1* sequence. The products were subsequently purified and recovered after electrophoresis. The target fragment was ligated to the digested vector, and the 10 μL *PoEP1* gene ligation system was set up as follows: 2 μL of gene *PoEP1* fragment, 3 μL of linearized plasmid, 4 μL of 2.5× *OK Clon* Master Mix, and 1 μL of ddH_2_O (Accurate Biology, Changsha, China). After ligation at 50 °C for 10 min, the DH5α competent *E. coli* cells were transformed, identified by colony PCR, and sent to Sangong for sequencing verification. The overexpression vector was named pCAMBIA2300-*PoEP1*. The constructed overexpression vector pCambia2300-*PoEP1* was transformed into the *Agrobacterium tumefaciens* strain GV3101 by the freeze–thaw transformation method. It was cultured in an LB solid medium containing kanamycin and rifampicin for 48 h. Monoclonal colonies were screened and verified by PCR, and the *Agrobacterium tumefaciens* successfully transferred into the recombinant vector was used for the later transgenic test in tree peony.

### 4.6. Subcellular Localization of PoEP1

Plant-mPLoc (http://www.csbio.sjtu.edu.cn/bioinf/plant-multi/, accessed on 6 November 2023) was used to predict the subcellular localization of *PoEP1* encoded proteins. PCR amplification of the CDS sequence of *PoEP1* without a termination codon was performed, which was then inserted into the vector pCAMBIA2300-GFP through a homologous recombination reaction. After spot-picking and PCR detection of the bacterial solution, the positive clone was sent for sequencing, and the correct bacterial solution was named pCAMBIA2300-*PoEP1*-GFP. The positive clone plasmid with correct sequencing was extracted and transformed into *Agrobacterium tumefaciens* GV3101 by the freeze–thaw method. The *Agrobacterium tumefaciens* solution of the *PoEP1* gene was transferred to a fresh LB liquid medium containing rifampicin and kanamycin for shaking propagation. The sample was centrifuged at 6000 rpm for 6 min, the supernatant was discarded, and the bacteria was resuspended 1–2 more times. Finally, the OD_600_ of the bacterial solution was adjusted to 0.4–0.6 and left to stand for 3 h in the dark and at room temperature. Using a 1 mL syringe, the resuspension was aspirated and injected into the back of a 6-week-old tobacco (*Nicotiana benthamiana*) leaf until the solution filled the entire leaf. The tobacco was then left in the dark overnight. Generally, the expression level is the highest 2–3 days after injection. The infected area was cut, the epidermis was cut, and the fluorescence signal was observed by laser scanning confocal microscopy. pCAMBIA2300-GFP was used as a control.

### 4.7. Agrobacterium tumefaciens-Mediated Overexpression of Arabidopsis thaliana

The constructed overexpression vector pCAMBIA2300-*PoEP1* and empty vector pCAMBIA2300 were introduced into *Agrobacterium* GV3101 by the freeze–thaw transformation method. *Agrobacterium*-mediated floral dipping method was used for *Arabidopsis* transformation. The harvested seeds were screened on 1/2MS Kan-resistant medium to identify positive plants, which were then transplanted into soil suitable for cultivation. Kan resistance screening is necessary for every generation. The PCR amplification of the *Arabidopsis* T3 generation samples is conducted to identify positive plants and observe their phenotypes.

### 4.8. Agrobacterium tumefaciens-Mediated Transient Overexpression of Tree Peony

The *Agrobacterium tumefaciens* overnight culture was transferred to a 50 mL centrifuge tube and centrifuged at 6000 rpm for 6 min at room temperature. The medium was discarded and suspended in the infiltration buffer composed of 10 mM MgCl_2_, 200 mM acetosyringone, and 10 mM MES, followed by centrifugation at 6000 rpm for 10 min. The infiltration buffer was discarded. The pellet of *Agrobacterial* cells was suspended in an infiltration buffer to an OD_600_ of 0.7 and placed at 28 °C for 3 h in the dark to prepare for transformation. Using ‘Luoyanghong’ fresh-cut flowers of the CE stage as the infection objects, each group had five replicates. The petals of the tree peony cut flower plant were immersed in the *Agrobacterium tumefaciens* bacterial solution resuspended with infiltration buffer, and infection with suction and a vacuum pump at 0.5 MPa for 20 min was performed. After completion of the infection treatment, the infected cut flower plants were taken out, rinsed in distilled water to wash off the bacterial solution, and inserted into a bottle container filled with distilled water. This was placed at 8 °C for 3 days, balanced at 23 °C for 1 day. Then, observe the flowers every 6 h as they develop, recording and sampling based on their growth stages and phenotype.

### 4.9. Data Analysis

SPSS 19.0 software was used to analyze all the data. The independent-sample t-test was used to detect the differences between the data of each group, and the Duncan test was used to detect the differences between more than two groups. A difference was considered to be significant when *p* < 0.05. The results of the analysis were graphed by Origin2022.

## 5. Conclusions

In this study, according to the analysis of the transcriptome and degradation group data in the laboratory, we found *PoEP1* and hypothesized that this gene is involved in the flowering-stage regulation of the tree peony. The ORF coding region of *PoEP1* was successfully cloned for the first time and subjected to bioinformatics analysis. The *PoEP1* was transformed into *Arabidopsis* and the tree peony; the function of *PoEP1* was explored and verified. The result indicated that *PoEP1* could advance plants’ flowering and senescence and shorten the flowering time of plants. The results of this study provide a theoretical basis for exploring the role of *PoEP1* in the regulation of the tree peony’s flowering stage, providing a theoretical and experimental reference for the study of the flowering mechanism of the tree peony. Furthermore, this lays a foundation for improving the ornamental and economic value of the tree peony.

## Figures and Tables

**Table 1:** Primer information.

Primer	Forward Sequence	Reverse Sequence	Usage
*EF1α*	CCGCCAGAGAGGCTGCTAAT	GCAATGTGGGAAGTGTGGCA	Internal reference genes
qPCR-*PoEP1*	ACCACCCCAAATGCTTACAC	CAGCAGAGTCAAGCAGAACCA	Real-time fluorescence quantification
pTOPO-*PoEP1*	CTGAGAACCAGACTTTCCATTT	CCCAATAAATAGAATCTCTCCTA	Gene cloning
pCAMBIA2300-*PoEP1*	CGGGGATCCTCTAGAGTCGACATGCTGAGCATCTTTAGTTCTCCA	AGGGCATGCCTGCAGGTCGACTTAATTGGATACCTTGATATAAGCCAC	Construct overexpression vector
pCAMBIA2300-*PoEP1*-GFP	CGGGGATCCTCTAGAGTCGACATGCTGAGCATCTTTAGTTCTCCA	TTCTCCTTTGCCCATGTCGACATTGGATACCTTGATATAAGCCACG	Constructing subcellular localization vectors

## Data Availability

The data supporting the results are already mentioned in the main text.

## Supplementary Materials

The following supporting information can be downloaded at https://www.mdpi.com/article/10.3390/plants13121642/s1: Figure S1. Nucleotide sequence and its encoded amino acid sequence.
