# Supplementary figures and images for: Functional Characterization of PoEP1 in Regulating the Flowering Stage of Tree Peony
Source: Plants (Basel). 2024 Jun 14;13(12):1642. doi: 10.3390/plants13121642 (PMC11207526; doi:10.3390/plants13121642)

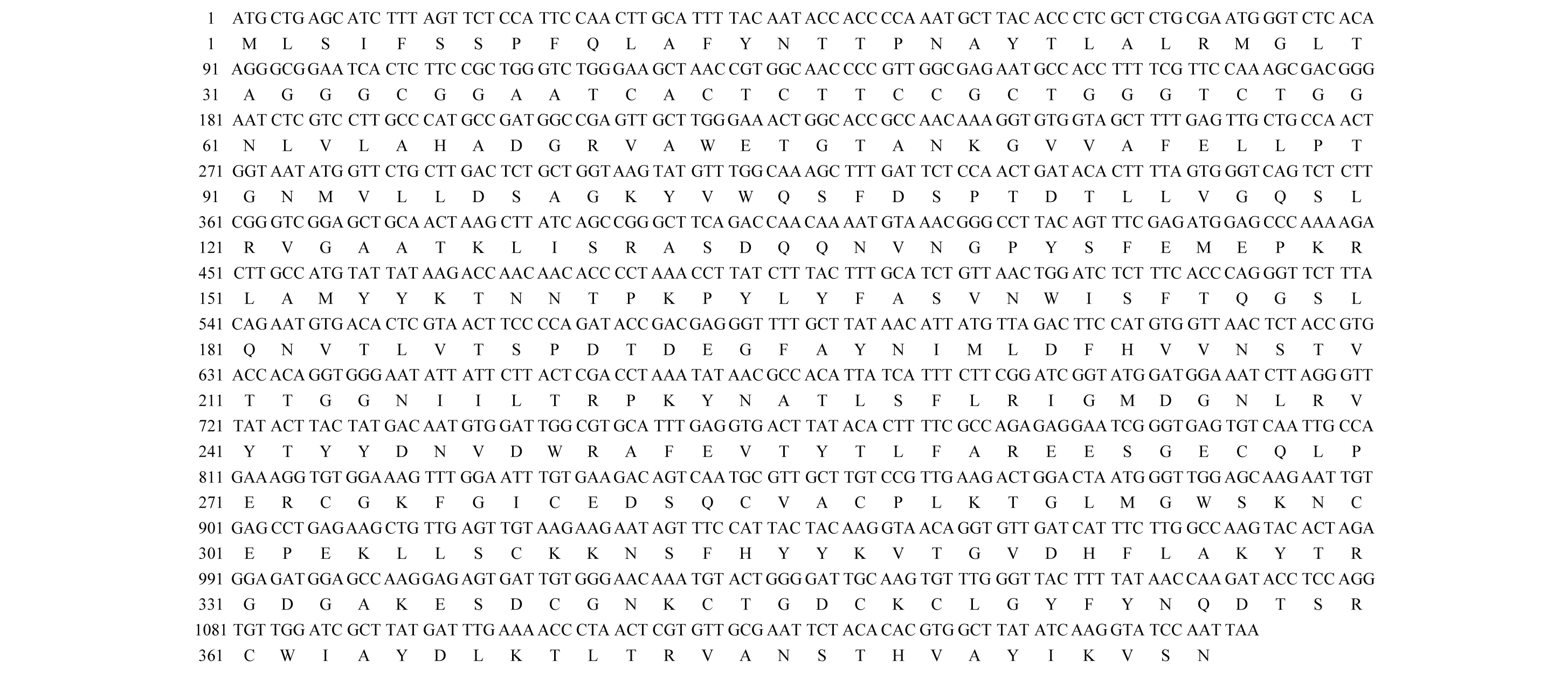

Supplement: Supplementary file 1 [file plants-13-01642-s001.zip › Figrue S1.tif]
